# A Practical Treatise on Foreign Bodies in the Air Passages

**Published:** 1855-04

**Authors:** 


ART. V.—*A Practical Treatise on Foreign Bodies in the Air Passages.* By S. D. Gross, M. D., Professor of Surgery in the University of Louisville; Member of the American Philosophical Society; Author of "Elements of Pathological Anatomy;" a "Treatise of Diseases of the Urinary Organs," etc., etc., etc. With Illustrations. Philadelphia: Blanchard and Lea. 1854.

The motto in this book expresses its character. "In an inquiry of this kind, in fact, individual experience amounts to nothing; collective experience is everything."

Accordingly this is a clinical report, its deductions being derived from the histories of cases from every attainable source. Surgical authors, isolated reports in medical periodicals, and modern surgeons, "blend their common toil" to make a book which exhausts the subject, and must forever remain the standard work on the management of this accident. Let not the reader look upon this as a "mere compilation." It would have been much easier for Prof. Gross to have written a "purely original work," based wholly upon "his own observation." But no single surgeon has seen enough of this accident to give character to his opinions, and "collective experience" has hitherto been wanting.

Prof. Gross has gathered in every field; and while he renders just acknowledgments to the numerous surgeons who have assisted his labor, that labor has not been the less tedious or onerous. No one, who has never attempted the analysis of a large number of medical cases, can know anything of the labor and tact required to mould them into intelligible and useful form.

A book like this—a book of facts—presents few salient points for the reviewer's grasp. We can only give an outline of the management recommended in the "general summary," at the close of the volume, and advise every one who has confidence enough in us to buy on our say-so, to possess himself of "Gross on Foreign Bodies" without delay.

The author objects to the use of all errhines, emetics, or of spontaneous efforts at expulsion of the foreign body, or of inversion or succession of the patient's body, as means which, though in rare instances successful, greatly endanger the sufferer. He advises bronchotomy, as without it the patient is at any time liable to suffocation from spasm of the glottis, and as the only reliable means of removing the intruder; and he would have a large opening, with many other matters of practical interest, for which we refer the reader to the volume itself.

For sale by MILLER, ORTON & MULLIGAN. H.

